# Efficacy of Interventions Based on the Use of Information and Communication Technologies for the Promotion of Active Aging

**DOI:** 10.3390/ijerph19031534

**Published:** 2022-01-29

**Authors:** Álvaro Astasio-Picado, Paula Cobos-Moreno, Beatriz Gómez-Martín, Lorena Verdú-Garcés, María del Carmen Zabala-Baños

**Affiliations:** 1 Nursing and Physiotherapy Department, Faculty of Health Sciences, University of Castilla-La Mancha, Real Fábrica de Sedas, s/n., 45600 Toledo, Spain; lorena.verdu@alu.uclm.es (L.V.-G.); Carmen.Zabala@uclm.es (M.d.C.Z.-B.); 2 Nursing Department, University of Extremadura, 10600 Cáceres, Spain; pacobosm@unex.es (P.C.-M.); bgm@unex.es (B.G.-M.)

**Keywords:** Tics, mobile applications, serious games, internet, virtual reality, home automation, active aging, elderly, gerontechnology, review

## Abstract

Nowadays, the study of how new media and technologies can be used to carry out health education by bringing these tools closer to the elderly population is interesting. It is a way of offering them access not only to greater knowledge, but to greater communication and relationship with their surroundings, a range of new possibilities and resources at their disposal that also represent a way to reduce the generation gap and bring them closer to the rest of the community. Objectives: to evaluate and analyze the studies that show the efficacy of interventions based on the use of information and communication technologies for the promotion of active aging in people older than or equal to 65 years who live in the community. Data sources, study eligibility criteria: the search for the articles was carried out from January 2012 to March 2021, in 6 databases (Pubmed, Cochrane Library, Scopus, Scielo, Google Academic and WOS) based on the clinical question, using the keywords derived from the DeCS and MeSH thesauri, combined with the Boolean operators “AND”, “NOT” and “OR”. The search was limited to publications from the last 9 years, in English and Spanish. Results: after applying the selection criteria and evaluating the quality of the methodology, 7.91% (*n* = 17) of the 215 results were included with filters: 7 systematic review, 5 of Cohorts and 5 of Randomized Controlled Trial. Conclusions and implications of key findings: the use of communication technologies reduces the feeling of loneliness, as well as the use of virtual reality to exercise, train memory or perform rehabilitation. The most difficult barrier to overcome is the prior ignorance of the majority of the elderly to the technology that is overcome by working as a team throughout the community, especially in the health and educational sector, as well as the family or social nucleus.

## 1. Introduction

In Spain, with a population of more than 47 million inhabitants, the population over 65 years of age corresponds to 19.58% of the total, compared to 7.62% corresponding to its birth rate. This translates into around 57,000 more deaths than births per year, which produces a contraction and aging of the Spanish population [1]. Life expectancy in this country is very high compared to other countries, the average being 83.5 years. This data can vary according to the region and be slightly higher in women than in men, reflected in the population pyramid [2].

Of almost 20% of the population over 65 years of age, more than 30% reach this age with moderate physical dependence. Meanwhile, 42% present pathologies of some kind, either cardiovascular (65%) or cognitive (43%), which are accentuated as individual ages [1,2]. For this reason, the term “healthy life expectancy” began to be used, to take into account not only the years lived, but also those in which good health and independence were maintained. In 2016, healthy life expectancy was 53.7 years for men and 44 for women [1,3].

The World Health Organization (WHO) defines active aging as the “process of optimizing opportunities for health, participation and safety with the aim of improving the quality of life as people age, and it is applicable not only to the individual, but to the entire community” [4]. In other words, active aging consists of taking advantage of all the available resources to maintain or increase health to an optimal level for as long as possible when people age, to achieve the best possible quality of life, something that will also affect the community [4,5].

The term “gerontechnology” was used for the first time in 1989, coined by Jan Graafmans, which refers to the relationship between aging and technology, aiming to contribute to the reduction of the problems of the elderly, which lead to aging transitions supporting compliance with the central strategy [6]. Within this concept, there are three main areas: technology for independent people, assistive technology and technology for communication and leisure; and three main barriers: decreased cognitive functions, accessibility, and lack of knowledge about technology [6,7]. These limitations and difficulties faced by this population segment in their daily lives, from basic activities at home to more complex activities such as transportation, have been increasing in recent years. These difficulties can be solved or diminished through the use of medical devices and/or medicine technologies resulting from gerontechnological investigations [8].

Today, the most downloaded and used mobile applications by the study group are those related to health, entertainment and social interaction and administrative tasks or transactions. Although its use is more and more frequent, fear and insecurity still appear, coming from the ignorance that some still feel [8]. But technology goes much further than downloading an app to train memory. Currently, it is possible to monitor a patient in real time in their own home, being able to remotely take constants and adapt the environment according to their needs; or train stunted limbs through virtual reality games. The possibilities for older people to benefit from the advances of this time are enormous, but it is in everyone’s hands to bring them the tools to access them [8,9].

The general objective of this work is to analyze the studies that demonstrate interventions based on the use of information and communication technologies (ICT) for the promotion of active aging in people older than or equal to 65 years living in the community.

## 2. Materials and Methods

This work was prepared using a qualitative systematic review in accordance with the Cochrane recommendations for qualitative evidence synthesis [10] and the PRISMA 2020 recommendations for systematic reviews [11]. The search was carried out in the following databases: Pubmed, Cochrane Library, Scopus, Scielo, Google Academic and WOS. To find the best possible scientific evidence, a series of inclusion and exclusion criteria were applied.

The keywords for this review were: Tics, mobile applications; serious games; internet; virtual reality; home automation; active aging; elderly; gerontechnology; review. These have been validated by DeCS and MeSH. Once selected, the corresponding Boolean operators were used: AND/OR, as well as the necessary parentheses and quotation marks. The criteria that have been taken into account for the selection of the relevant studies are the following. Inclusion criteria: the period between January 2012 to March 2021; studies based on interventions carried out through ICTs or other digital media; studies aimed at the target population, that is, people aged 65 years or older; studies that address issues of common chronic diseases of old age, since prevention must begin before the disease appears; the selected studies must have scientific evidence and be published in corroborated databases; Spanish or English language; studies that show the benefits or harms of the use of new technologies to achieve active aging. Exclusion criteria: articles prior to 2012; language: not English not Spanish; studies in which the population was under 65 years of age; studies that do not provide scientific evidence justified by the level of indexing of articles in journals according to the latest certainties.

For the methodological evaluation of the individual studies and the detection of possible biases, the evaluation is carried out using the “PEDro Evaluation Scale”. This scale consists of 11 items, providing one point for each item that is met. The articles that obtain a score of 9–10 points will have an excellent quality, those between 6–8 points will have a good quality, those that obtain 4–5 points will have an intermediate quality, and, finally, those articles that obtain less than 4 points will have a poor methodological quality articles [12].

The Scottish Intercollegiate Guidelines Network (SIGN) classification will be used in the data analysis and assessment of the levels of evidence, which focuses on the quantitative analysis of systematic reviews and on the reduction of systematic error. Although it takes into account the quality of the methodology, it does not assess the scientific or technological reality of the recommendations [13].

## 3. Results

**Figure 1:**
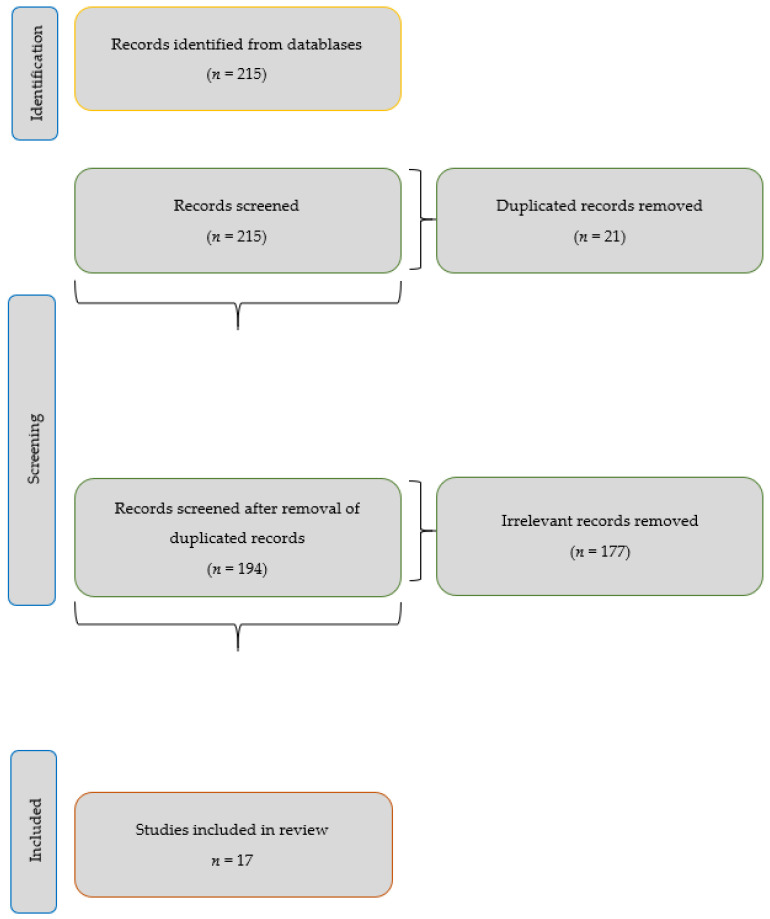
Flow diagram.

The review question was constructed following the PICO format (Population/patient, Intervention, Comparator and Outcomes/Outcomes). Detailed as “People over or equal to 65 years of age who live in the community (P), Interventions that use new information and communication technologies (ICTs) (I), Interventions that do not use the new usual analog information and communication technologies (C), Achievement of higher quality active aging (O) (Figure 1).

Below is a table that shows the search strategy used to select the 17 articles selected from the 6 databases and following the criteria of identified studies, duplicate studies, title, abstract, full text and valid studies of a definitive nature (Table 1). The total number of valid articles is summarized in Appendix A. 

### 3.1. Analysis of the Barriers and Limitations of the Elderly to Access Technologies

Mobile health applications produce beneficial effects in the elderly population [14,15,16,17,18]. This is demonstrated by the decrease in sedentary lifestyle and the increase in physical activity and fitness in trials of less than 3 months and an increase in physical activity in those older than 6 months [14,16]. The “Falls Sensei” game provides education on extrinsic risk factors for falls, as well as raising awareness and facilitating hazard detection at home [15]. Thus, the collaborative game mode in exergames promotes social participation and improves empathy and interaction among participants [15,16]. In another study the experimental group underwent virtual reality therapy 30 min a day, 3 times a week, for 6 weeks. An improvement in static and dynamic equilibrium was observed [16,17]. Home therapy, although it took less time than expected (57.9%), obtained excellent results in terms of suitability and improvement of rhythmic skills [18].

### 3.2. Study of the Temporary Dedication of the Elderly to the Use of New Technologies

Different studies carried out through an experimental group, which carried out the exergame-based therapy, showed better results in coordination, movement speed and dexterity in relation to the control group [19,20,21,22]. The group of users with the lowest chronological age being the ones that use the networks the most, as well as those with the best cognitive state [19,20]. Other studies show that the biggest gap continues to be anxiety about the use of technology, something that can be compensated with training and experience [20,21,22]. Older people find the use of the Internet useful to learn more about their disease or as an aid to improve their adherence and administration of medication [19,21]. Even so, the main disadvantage is that technology is still stigmatized as difficult to use [21,22,23]. Study participants used the mobile phone as a means of improving their adherence to cardiovascular disease prevention treatment for 12 months [22,23]. In addition, a study of video calls revealed that they have a minimal effect on feelings of loneliness or quality of life, but a decrease in depression symptoms is observed at 12 months compared to those who did not receive video calls [23,24]. Another study showed the benefits of the use of smart technology in self-care, quality of life and physical activity in a period of up to 6 months. Despite this, there was no evidence of long-term use, although it is possible to maintain it over time in those most interested in the technology [25,26,27]. In other studies, participants were satisfied with the monitoring of mobile applications. Its use was perceived as easy and useful for the administration of medications at home, thus improving adherence [26,27]. The experimental group that received follow-up through mobile applications showed a greater reduction and control of their BP and a greater adherence to antihypertensive medication [26,27,28]. Telecare has also been reported to help control signs and symptoms, weight, and self-care. As a consequence, greater adherence to treatment and a reduction in the number of hospitalizations and mortality are achieved [27,28].

### 3.3. Study of the Individual’s Perception Regarding the Use of New Technologies

Older people are increasingly interested in knowing the Internet, as well as its use and operation [16,18,27,28]; especially for information on health, diseases, hospitals and health centers, current news or diets [26,27,28]. In addition, it is frequently used for family contacts and administrative purposes [28,29]. In another study, the experimental group that received control through the telemedicine system showed significant improvement at 3 and 6 months compared to the control group [30]. Trials of self-monitoring of blood pressure by mobile phone reported modest beneficial effects [23,28]. Similarly, one trial with a low risk of bias reported modest reductions in LDL cholesterol, but no beneficial effects on blood pressure [23,29].

### 3.4. Methodological Evaluation of the Studies and Data Analysis and Evaluation of Evidence

The evaluation is carried out by means of the “PEDro Evaluation Scale”, to the 5 randomized clinical studies, on the scale of 11 items, 1 being an intermediate evaluation study (4–5 points), 3 of a good evaluation (6–8 points) and 1 for excellent evaluation (9–10 points) (Table 2) [12].

The Scottish Intercollegiate Guidelines Network (SIGN) classification is used in the data analysis and evaluation of the levels of evidence, assessing the 7 systematic reviews with category 2 and 3, and the 5 cohort studies, assessing 2 as 2++ and 3 as 2+ (Table 3) [13].

Regarding the methodological evaluation of the studies and data analysis and evaluation of evidence, the evaluation was carried out using the PEDro Evaluation Scale, obtaining an intermediate evaluation of good and excellent [12]. In addition, the SIGN, a tool used in the data analysis and evaluation of the levels of evidence, evaluating the 7 systematic reviews with categories 2 and 3evaluating 2 as 2++ and, and the 5 cohort studies, 3 as 2+ [13].

## 4. Discussion

In the article “Older people and the internet: the web as a source of opportunities for active aging” by Lorente Barroso et al. [29], it is observed how the use of the internet by older people is increasing and focuses on especially in obtaining information about your health and diseases, medicines and hospitals, diets, transactions or related to family and friends. On the contrary, although they consider its use very useful, according to the study “Analysis of the elderly consumer in the use of sanitary applications or APPS” by Mañas Viniegra et al. [21], they are still reluctant because there are processes that consider it difficult or unsafe. Therefore, in most cases they usually need help from third parties to perform fairly simple operations. These fears and insecurities appear especially in the elderly or with cognitive problems, since, at an earlier age, more networks use a better cognitive state, a greater number of different networks, as the study “From the digital gap to the gap digital Psycho-digital: older people and social networks ”by Peral et al. [20].

In the beginning, when talking about new technologies and media, people tend to think mostly about how to have close relatives and loved ones. Therefore, this is one of the main applications that the elderly give it. According to the study “Video calls to reduce social isolation and loneliness in the elderly” by Noone et al. [24], this connection has positive effects in alleviating the signs and symptoms of depression in the long term. Despite this, the feeling of loneliness or isolation remains. This new way of relating also improves interpersonal relationships. In this way, empathy and social participation with others increases through collaborative games. These same games also provide cognitive training in aspects such as memory, as well indicated by the “Computer-based cognitive training for the maintenance of cognitive functionality in older people without cognitive impairment” Gates et al. [22], although these effects have only been observed in the short term, with no evidence that they are sustained over time.

Although this use of ICTs seems very simple and everyday, each time they have been refined and specified, so that today there are multiple mobile health apps that help the population to control and prevent their diseases.

The beginning of these apps was especially those dedicated to medication reminders that help to promote therapeutic adherence at home, as clearly explained in the articles “Telecare as a health strategy for the adherence of patients with heart failure—integrative review” by Ribeiro and Jesús et al. [28], “Mobile health applications for the management of primary hypertension: A multicenter, randomized, controlled trial” by Gong et al. [27] or “Mobile Apps for Increasing Treatment Adherence: Systematic Review” by Pérez Jover et al. [26] in whose articles it is clear how the use of mobile apps can contribute to maintaining BP and weight control, improving the quality of life of cardiac, diabetic or COPD patients, for example, since It is a simple way to control medication at home, avoiding forgetfulness or errors in the doses, improving the progress of the disease and reducing hospitalizations and mortality.

Although these authors agree on the benefits of ICTs and health applications, they do not specify how the interventions should be carried out, as mentioned in “Mobile Phone-Based Telemedicine Practice in Older Chinese Patients with Type 2 Diabetes Mellitus: Randomized Controlled Trial ”by Sun et al. [30] in which special importance is given to the time and use of the system and specifies that the needs are not the same in each subject; in the same way as mentioned in the study “Interventions provided by mobile phones to improve adherence to medication in patients to prevent cardiovascular diseases” by Palmer et al. [23], in which the results were only observed after 12 months of intervention. This is largely due to the importance that subjects know how to use the networks and commit to their use, but it is indisputable that self-care is very favorable if it is carried out under these premises, as reflected in the study “Intelligent technology for COPD self-care” by McCabe et al. [25].

Apart from these basic applications, there is more sophisticated technology that helps rehabilitation and physical training, improving physical condition avoiding sedentary lifestyle, as indicated in the study “Effects of Mobile Health App Interventions on Sedentary Time, Physical Activity, and Fitness in Older Adults: Systematic Review and Meta-Analysis” by Yerrakalva et al. [14], although long-term effects have not been proven. Its usefulness in improving coordination, balance or rhythmic attitudes has also been studied, as reflected in “Virtual-reality balance training with a video-game system improves dynamic balance in chronic stroke patients” by Cho et al. [17], “Home-based training of rhythmic skills with a serious game in Parkinson’s disease: Usability and acceptability” by Dauvergne et al. [18] coinciding with “Leap motion controlled video game-based therapy for upper limb rehabilitation in patients with Parkinson’s disease: a feasibility study” by Fernández González et al. [19], although this differs from the previous one in the importance of studying long-term effects.

If the range is also expanded, it can be seen that apart from the use of the internet and mobile apps, there are multiple other technologies available to our elders that can increase their quality of life, such as exergames, virtual reality, such as in the study “Falls Sensei: a serious 3D exploration game to enable the detection of extrinsic home fall hazards for older adults” by Money et al. [15], in which patients are placed in real contexts that allow them to determine future risks at home or through home automation, which allows remote control of the environment, adapting it to the patient’s needs and monitoring their vital signs from distance.

Although these new technologies still need to be perfected, their great utility in helping to maintain health and self-care is indisputable, giving them the independence and autonomy necessary to maintain or increase their quality of life. Although most of the studies analyzed show that these new tools are beneficial for the proposed objective, there are many gaps in terms of the knowledge that the elderly have about these new methods.

## 5. Conclusions

ICTs reduce the feeling of loneliness or helplessness in active aging. The use of virtual reality has been demonstrated for exercise, memory training or rehabilitation. To analyze the barriers and limitations of the elderly to access technologies, we conclude that the greatest inconvenience is the lack of knowledge about the technologies and the fear that this provokes, requiring teamwork from the entire community, especially from the health and educational sector as well as the family or social nucleus, always personalizing the teachings to the individual, giving them the necessary time to hit and miss. The main interests of this population segment are communication with relatives and friends or the use of social networks. Although they also use them for administrative or banking processes, as well as health applications, whether they are about food, pedometers, memory games or apps for monitoring diseases or leisure. Regarding the greatest fear for the use of these technologies is a possible inappropriate use of your data.

## Figures and Tables

**Table 1:** Databases consulted.

Item Criteria	Cochrane	Google Scholar	Medline/Pubmed	Scielo	Scopus	WOS	Total
Identified	53	78	39	14	12	19	215
Duplicates	4	7	3	4	2	1	21
Title	16	23	13	9	6	5	72
Abstract	14	19	12	7	4	4	60
Text complete	11	13	9	6	3	3	45
Valid	6	4	4	1	1	1	17

**Table 2:** PEDro Evaluation Scale [16,17,19,27,30].

Item Criteria	Pereira et al. 2019 [16]	Cho et al. 2012 [17]	Fernández González et al. 2019 [19]	Gong et al. 2020 [27]	Sun et al. 2019 [30]
The selection criteria are specified.	YES	YES	YES	YES	YES
Subjects were randomly assigned to groups.	YES	YES	YES	YES	YES
The assignment was hidden.	NO	YES	YES	NO	YES
The groups were similar with respect to the most important indicators.	YES	YES	YES	YES	YES
All subjects were blinded.	NO	NO	NO	NO	YES
All individuals who administered the therapy were blinded.	NO	NO	NO	NO	NO
All raters were blinded.	NO	NO	NO	NO	YES
At least one of the key results was obtained in more than 85% of the subjects.	YES	YES	YES	YES	YES
Results were presented for all subjects.	YES	YES	YES	NO	YES
Comparisons of at least one key outcome were obtained.	YES	YES	YES	YES	YES
The study provides point and variable measures of at least one key outcome.	YES	YES	YES	YES	YES
Result	7	8	8	5	9

**Table 3:** Scottish Intercollegiate Guidelines Network (SIGN) classification [14,15,16,17,18,19,20,21,22,23,24,25,26,27,28,29,30].

Item Criteria	Item Criteria Description	Article
Experimental (randomized controlled trials)	1 ++ Meta-analysis of RCTs and SR of high quality RCTs or RCTs with very low risk of bias.	Cho et al. 2012 [17]. Gong et al. 2020 [27].
	1 + Meta-analysis of RCTs and SR of well-done RCTs or RCTs with low risk of bias.	Pereira et al. 2019 [16]. Fernández González et al. 2019 [19].
	1.- Meta-analysis of RCTs and SR of RCTs, or RCTs with high risk of bias.	Sun et al. 2019 [30]
Observational analytics (cases and controls or cohorts)	2 ++ High-quality SR of case-control or cohort studies, or high-quality case-control or cohort studies with very low risk of confusion, bias or chance and a probability that the relationship is causal.	Money et al. 2019 [15]. Dauvergne et al. 2018 [18].
	2+ Well-done case-control or cohort studies with a low risk of confusion, bias, or chance and a moderate probability that the relationship is causal.	Mañas Viniegra 2015 [21]. Peral et al. 2015 [20]. Lorente Barroso et al. 2015 [29].
	2.Case-control or cohort studies with a high risk of confusion, bias or chance and a significant risk that the relationship is not causal.	Yerrakalva et al. 2019 [14].
Descriptive	3. Non-analytical studies, for example, case series or case descriptions.	Gates et al. 2019 [22]. Palmer et al. 2021 [23]. Noone et al. 2020 [24]. McCabe et al. 2017 [25]. Pérez-Jover et al. 2019 [26]. Ribeiro de Jesús et al. 2020 [28].
	4. Expert opinion.	-

## Abbreviations

WHO	World Health Organization.
ICTs	Communication technologies.
SIGN	Scottish Intercollegiate Guidelines Network.

## Appendix A

**Table A1:** Selected Scientific Articles Table [14,15,16,17,18,19,20,21,22,23,24,25,26,27,28,29,30].

Authors; Year	Type of Study	Patients	Conclusion
Yerrakalva et al., 2019 [14]	Systematic review and meta-analysis.	6 trials with 486 participants aged 68 years on average.	Mobile health apps produce beneficial effects such as decreased sedentary lifestyle and increased physical activity and fitness in trials of less than 3 months and increased physical activity in those of more than 6 months.
Money et al., 2019 [15]	Experimental study	15 elderly residents in the community.	The game “Falls Sensei” provided further education on extrinsic risk factors for falls, in addition to raising awareness and making it easier to detect hazards at home.
Pereira et al., 2019 [16]	Randomized clinical trial.	40 adults over 70 years old.	The collaborative game mode in exergames promotes social participation and improves empathy and interaction between participants.
Cho et al., 2012 [17]	Randomized clinical trial.	22 adults over 64 years old.	The experimental group underwent virtual reality therapy 30 min a day, 3 times a week, 6 weeks. An improvement in static and dynamic balance was observed.
Dauvergne et al., 2018 [18]	Experimental study.	16 adults with an average age of 65.	Home therapy, although it took less time than expected (57.9%), obtained excellent results in terms of suitability and improvement of rhythmic skills.
Fernández González et al., 2019 [19]	Randomized clinical trial	23 patients	The experimental group, which performed the therapy based on exergames, showed better results in coordination, speed of movements and dexterity in relation to the control group.
Peral et al., 2015 [20].	Observational study.	415 patients with a mean age of 63.6 years.	Those with a lower chronological age are the ones who use the networks the most, and those with better cognitive status are the ones who use the most different networks. The study showed that the biggest gap is still anxiety towards technology, something that can be compensated with training and experience.
Mañas Viniegra L. 2015 [21]	Observational study.	1065 participants over 65 years of age.	The results showed that the elderly consider the use of the internet useful to learn more about their disease or to help them improve their adherence and administration of medication; the downside is that they still stigmatize technology as difficult to use.
Gates et al., 2020 [22]	Systematic review.	8 RCTs with 1183 participants aged 65 years and over.	After 12 weeks of computer training, a better cognitive function was observed, especially in aspects such as memory, although there is no evidence on its long-term effects.
Palmer et al., 2021 [23]	Systematic review.	4 RCTs with 2429 participants.	The participants used the mobile phone as a means to improve their adherence to CD prevention treatment for 12 months. The results were slightly positive, although not in all trials.
Noone et al., 2020 [24]	Systematic review.	3 studies with 201 older adults.	The study revealed that video calls have a minimal effect on feelings of loneliness or quality of life, but a decrease in depression symptoms is observed at 12 months compared to those who did not receive video calls.
McCabe wt al., 2017 [25].	Systematic review.	3 RCTs with 557 participants with a mean age of 64 years.	The study showed benefits of the use of smart technology in self-care, quality of life and physical activity in a period of up to 6 months; there was no evidence of long-term use, although it is possible to maintain it over time in those most interested in technology.
Pérez-Jover et al., 2019 [26].	Systematic review.	11 studies of between 16 and 99 adult participants.	All participants were satisfied with the monitoring of mobile apps. Its use was perceived as easy and useful for the administration of medication at home, thus improving its adherence.
Gong et al., 2020 [27].	Randomized clinical trial.	480 adult participants.	The experimental group that received follow-up through mobile apps showed a greater reduction and control of their BP and a greater adherence to antihypertensive medication.
Ribeiro de Jesús et al., 2020 [28].	Integrative review.	12 articles with 6314 adult participants.	The study showed that telecare helps in the control of signs and symptoms, weight and self-care. As a consequence, greater adherence to treatment and a reduction in the number of hospitalizations and mortality is achieved.
Lorente Barroso et al., 2015 [29].	Observational study.	3 discussion groups made up of 5–6 participants between 56 and 81 years old.	Older people are increasingly interested in knowing the internet and how it works; especially for information on health, diseases, hospitals and health centers, current news or diets. In addition, it is frequently used to be in contact with family members and for administrative purposes.
Sun et al., 2019 [30].	Randomized controlled trial.	91 participants aged 65 years or older with DM2.	The experimental group that received control through the telemedicine system showed a significant improvement at 3 and 6 months compared to the control group.
